# Supplementary material for: DNA Methylation Changes and Phenotypic Adaptations Induced Repeated Extreme Altitude Exposure at 8848 Meters
Source: Int J Mol Sci. 2024 Nov 25;25(23):12652. doi: 10.3390/ijms252312652 (PMC11641581; doi:10.3390/ijms252312652)
Supplement: Supplementary file 1 [file ijms-25-12652-s001.zip › Supplement map legend.pdf]

Fig. S1. Effect of different times of RET environment on CpG and phenotype. A. The correlation between 15 phenotypes and 13 CpG as well as Climbing age was studied in a network using Spearman's method (P value < 0.05, |Coef| > 0.3). **B.** The number of CpGs significantly associated with the 15 phenotypes. **C.** The PcoA analysis was performed using 13 CpG to downscale 64 volunteers, which was able to distinguish RET from non-RET populations. **D.** The PcoA analysis was performed using 12 CpGs (excluding cg23325384) to downscale 64 volunteers, which were were able to distinguish between those with >6 times RET. **E.** The correlation between 15 physiological phenotypes of 64 volunteers (P value < 0.05, |Coef| > 0.3)**.**

Fig. S2 MP-SSN network CpGs cross-omics cross-queue validation. A-F. Status of LIPN, PLCH1, EMR1, STX5 genes in the AltitudeOmics：(1) Significant changes in gene expression values at different stages of hypoxia exposure. (2) Significant changes in Beta values of CpG at different stages of hypoxic exposure.

Fig. S3 Features of the MP-SSN network in populations with differences in oxygen saturation. A.B. Differences in phenotypes and CpGs between high and low adapted groups (CpG was tested by F-test and Phenotype by T-test). **C.D.** Phenotypic and CpG differences between High and Low at 5 times RET (*** P < 0.001).

Fig. S4 Based on the height of SP O2 as a group, we explore the changes in 13 CP G and related phenotypes in the packets of CA. A. High adaptability. **B.** Low adaptability.

Fig. S5. Cell infiltration status score. A. Distribution of cell infiltration ratios of Non-RET and RET. B. Cells with significant differences between Non-RET and RET (based on T test)
