# Supplementary figures and images for: DNA Methylation Changes and Phenotypic Adaptations Induced Repeated Extreme Altitude Exposure at 8848 Meters
Source: Int J Mol Sci. 2024 Nov 25;25(23):12652. doi: 10.3390/ijms252312652 (PMC11641581; doi:10.3390/ijms252312652)

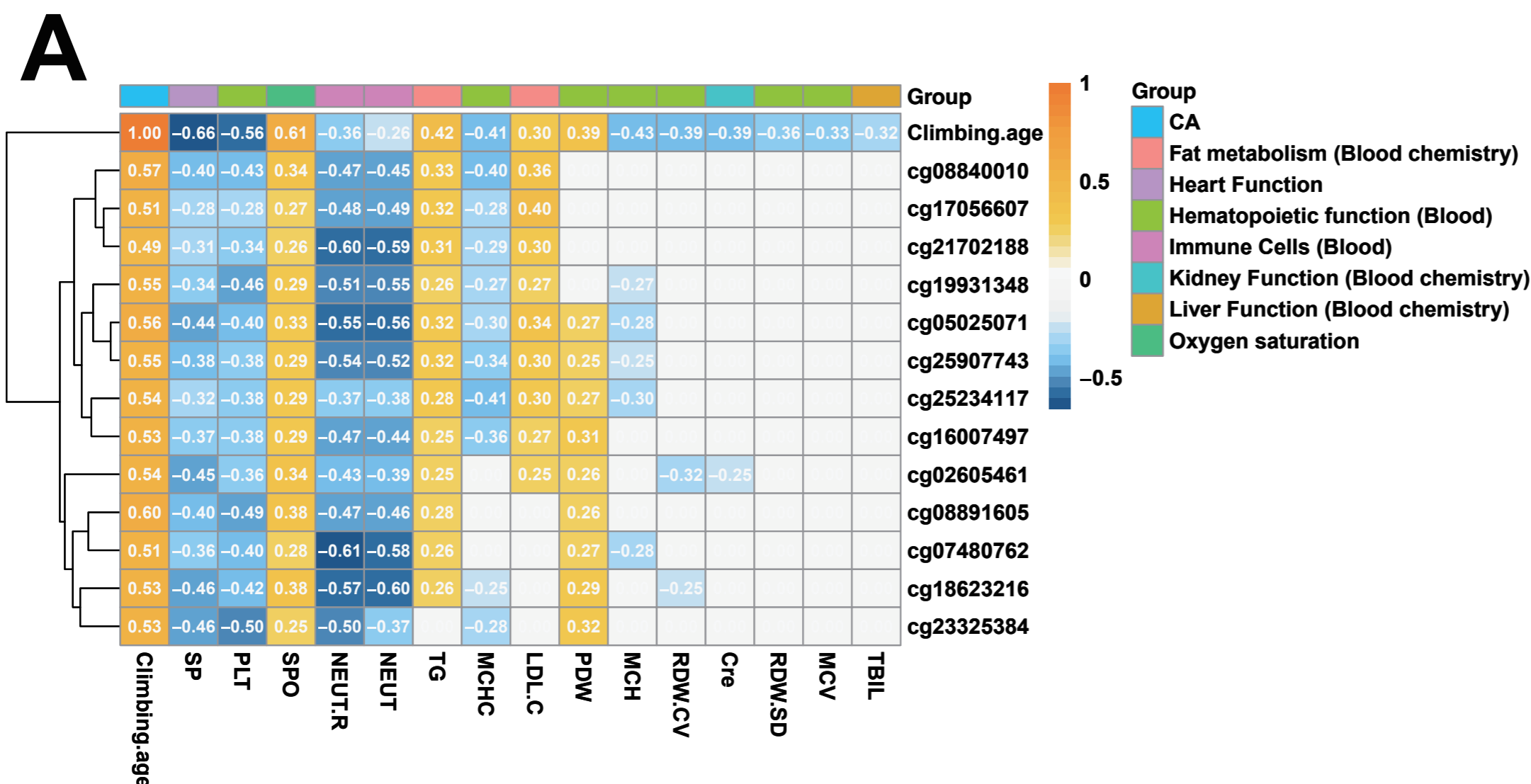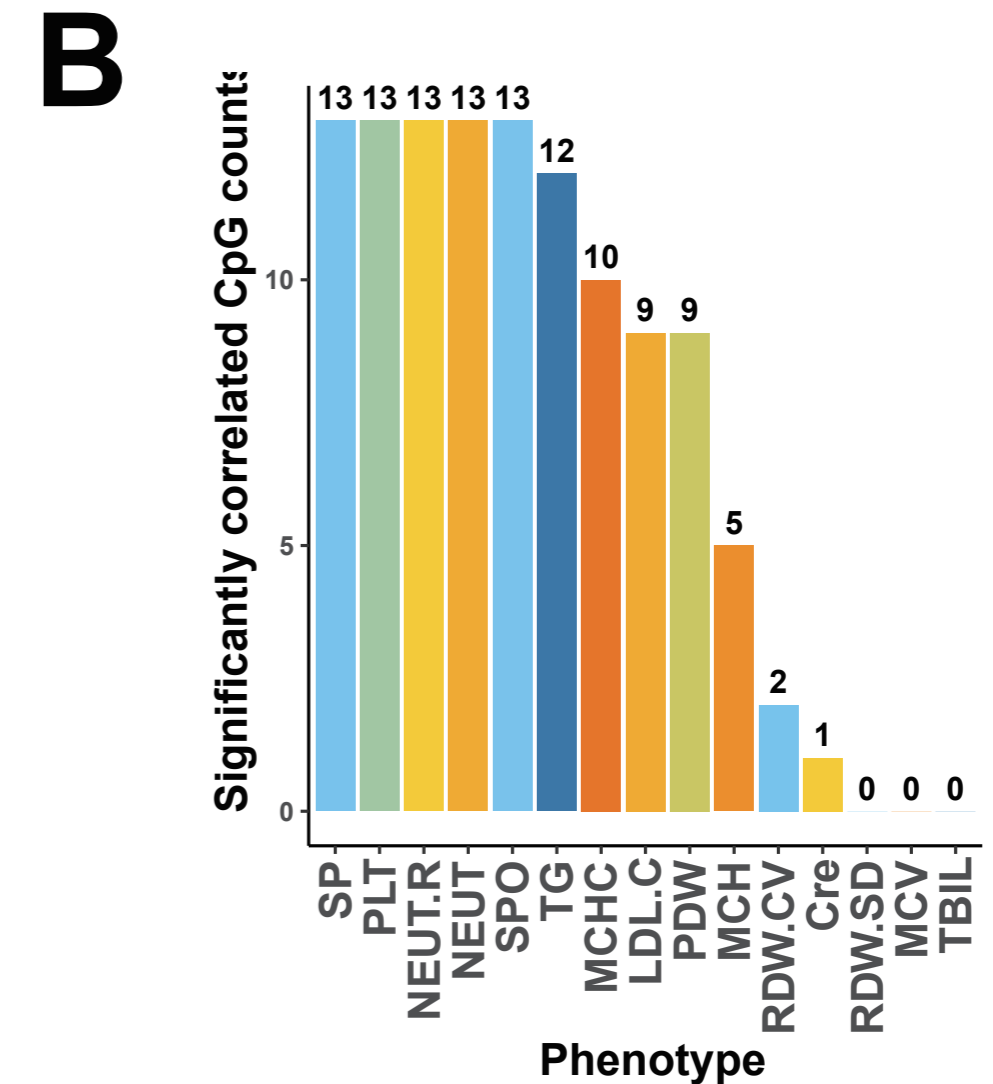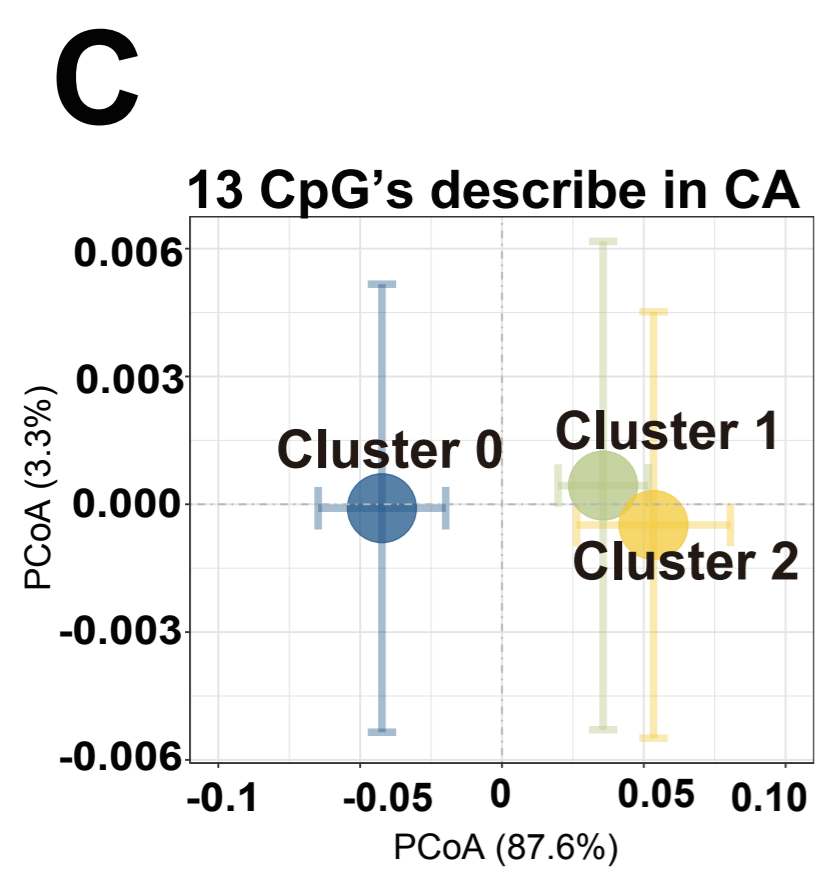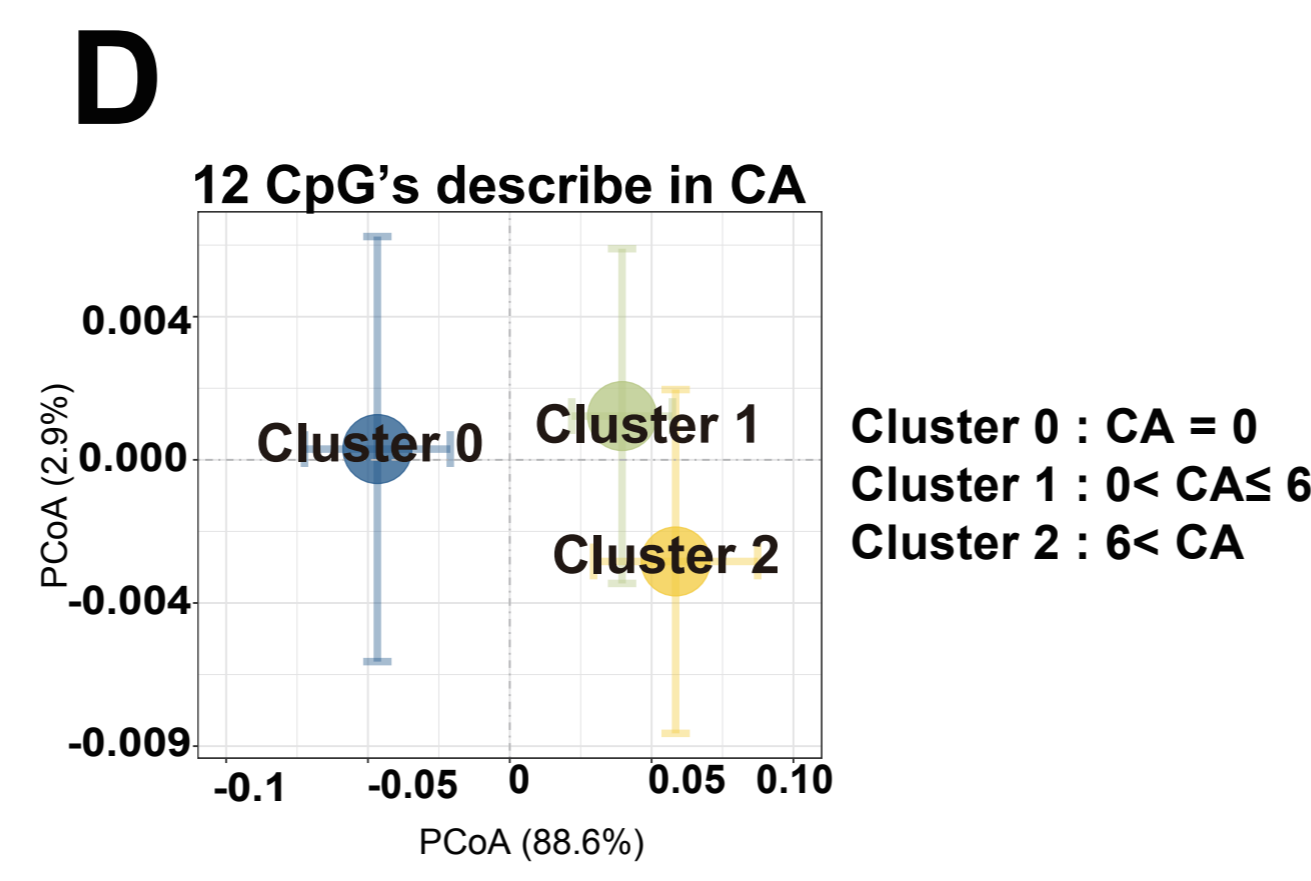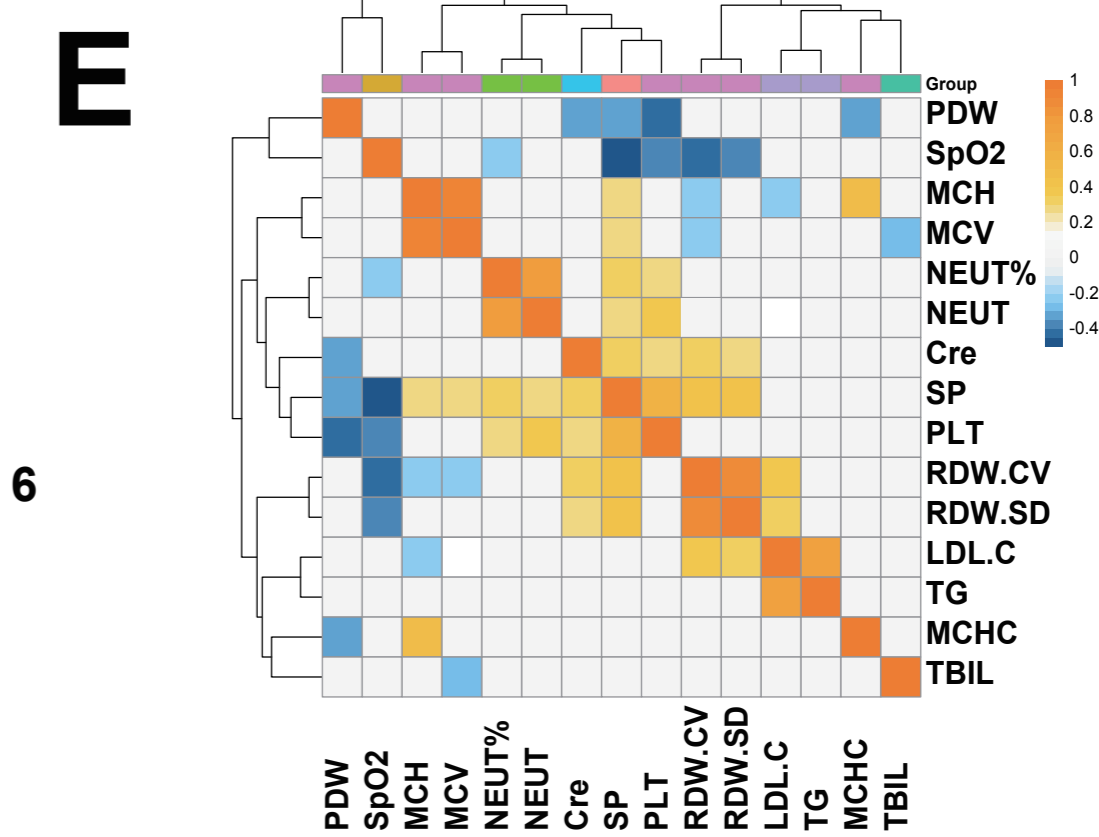

Supplement: Supplementary file 1 [file ijms-25-12652-s001.zip › FIg S1.pdf]

## A LIPN eQTM

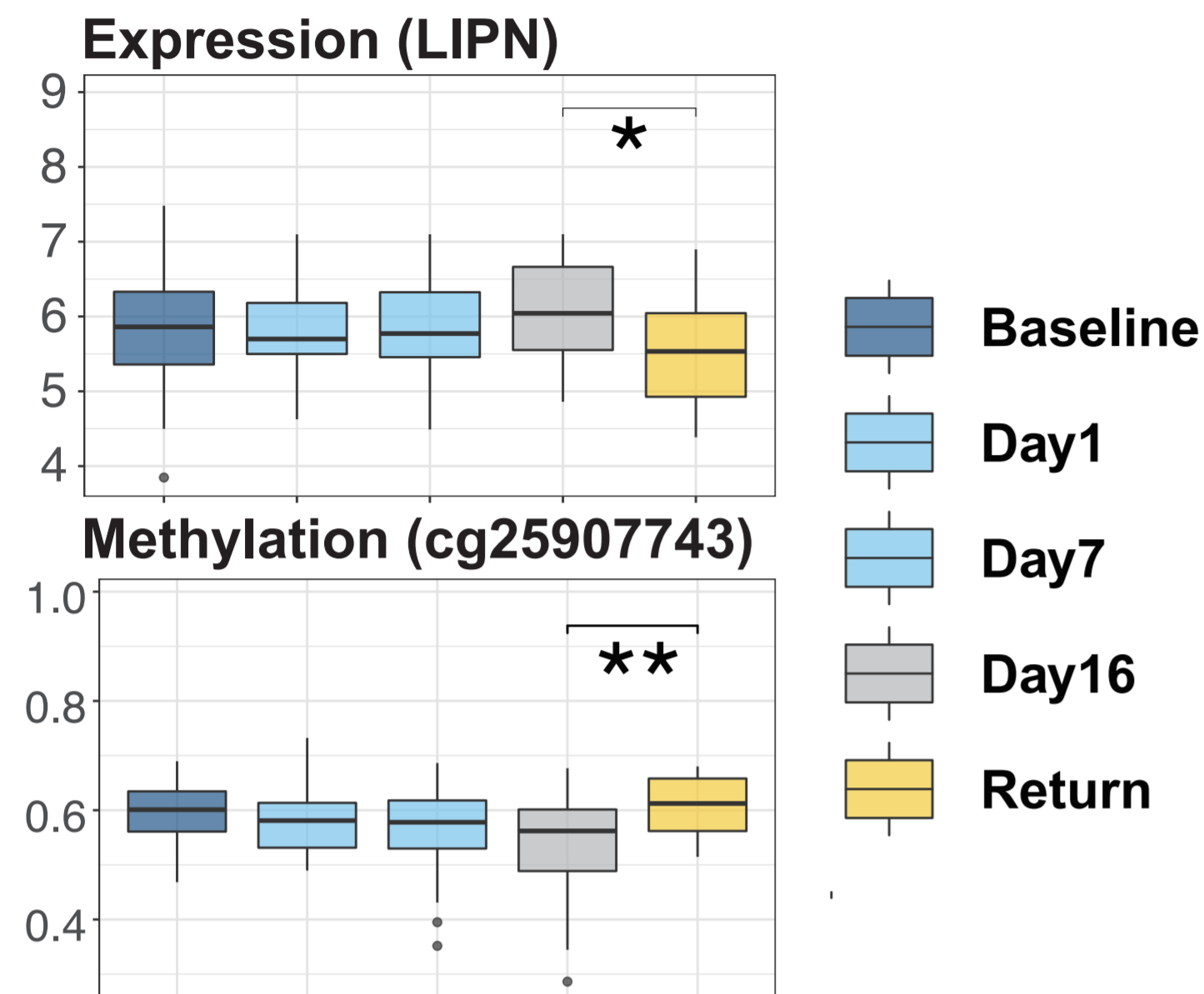

## B PLCH1 eQTM

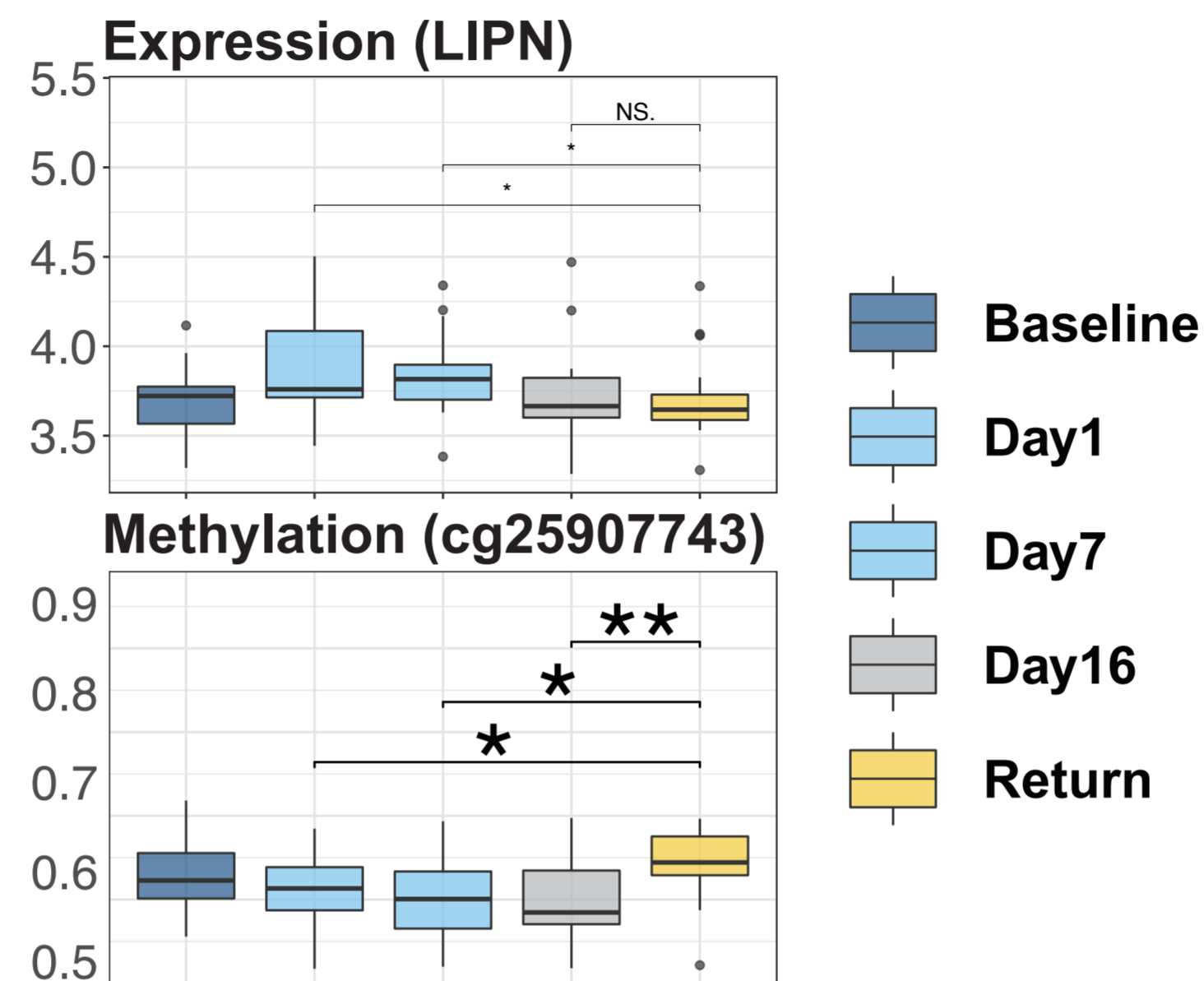

## C PLCH1 eQTM

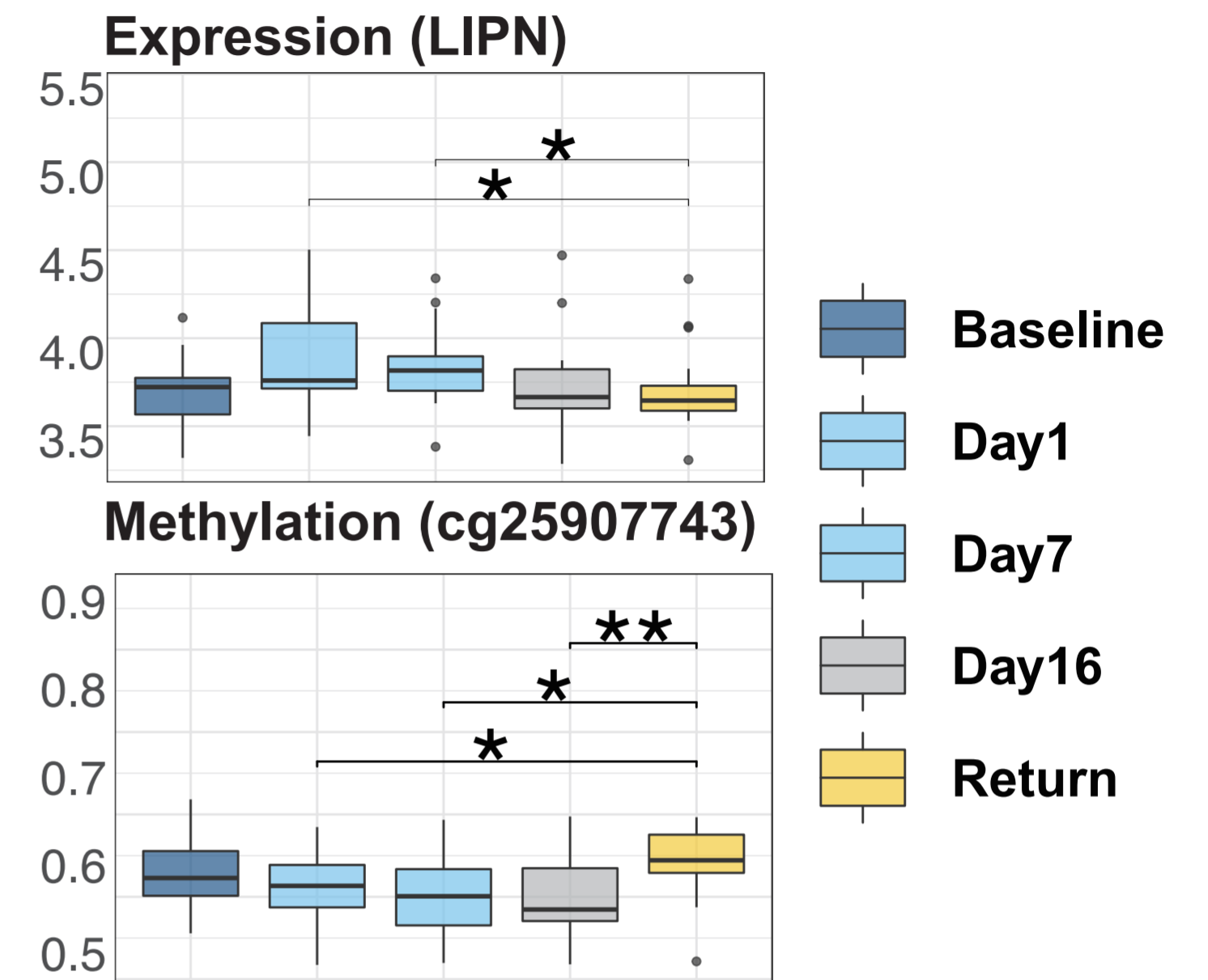

## D PLCH1 eQTM

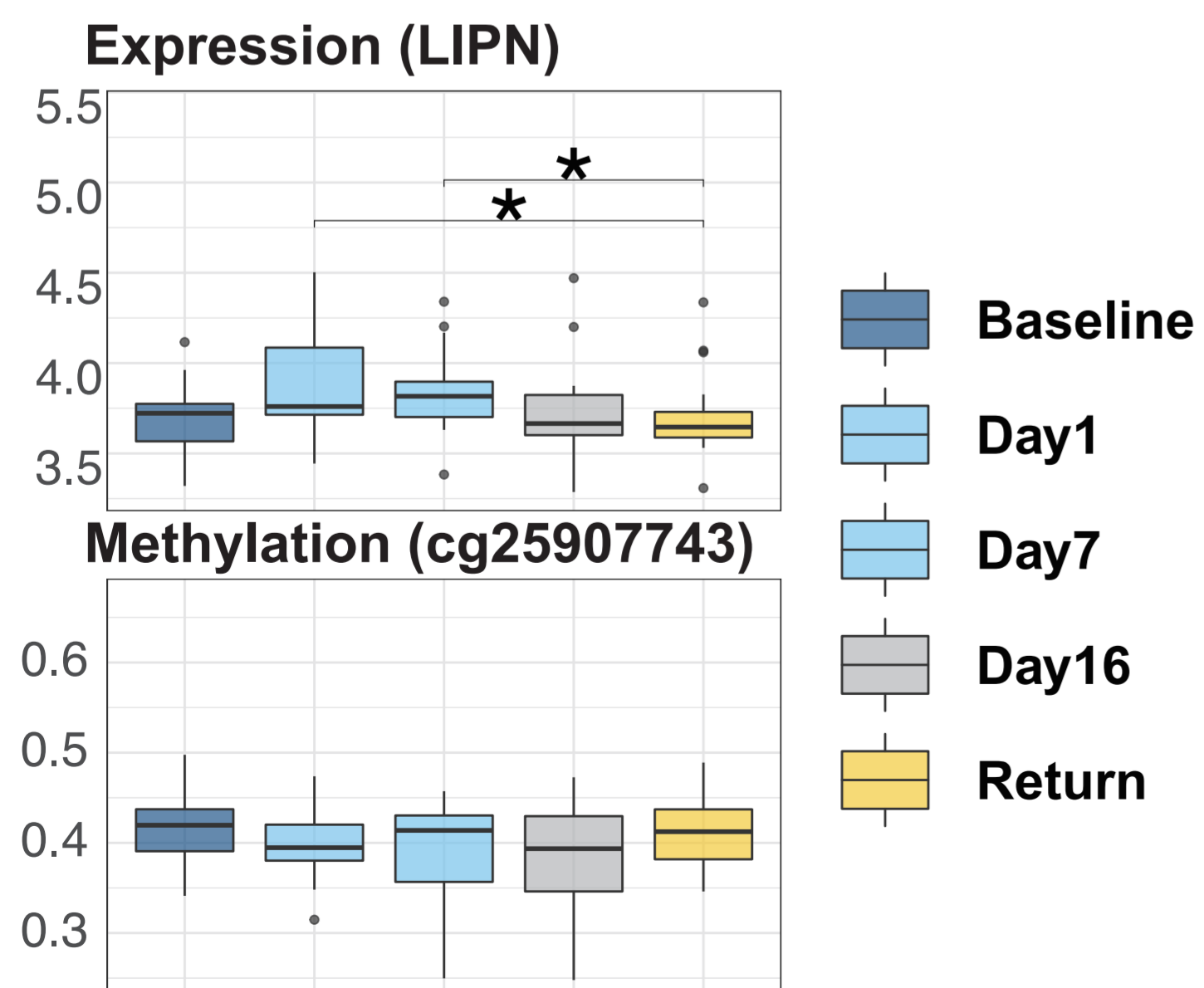

## E EMR1 eQTM

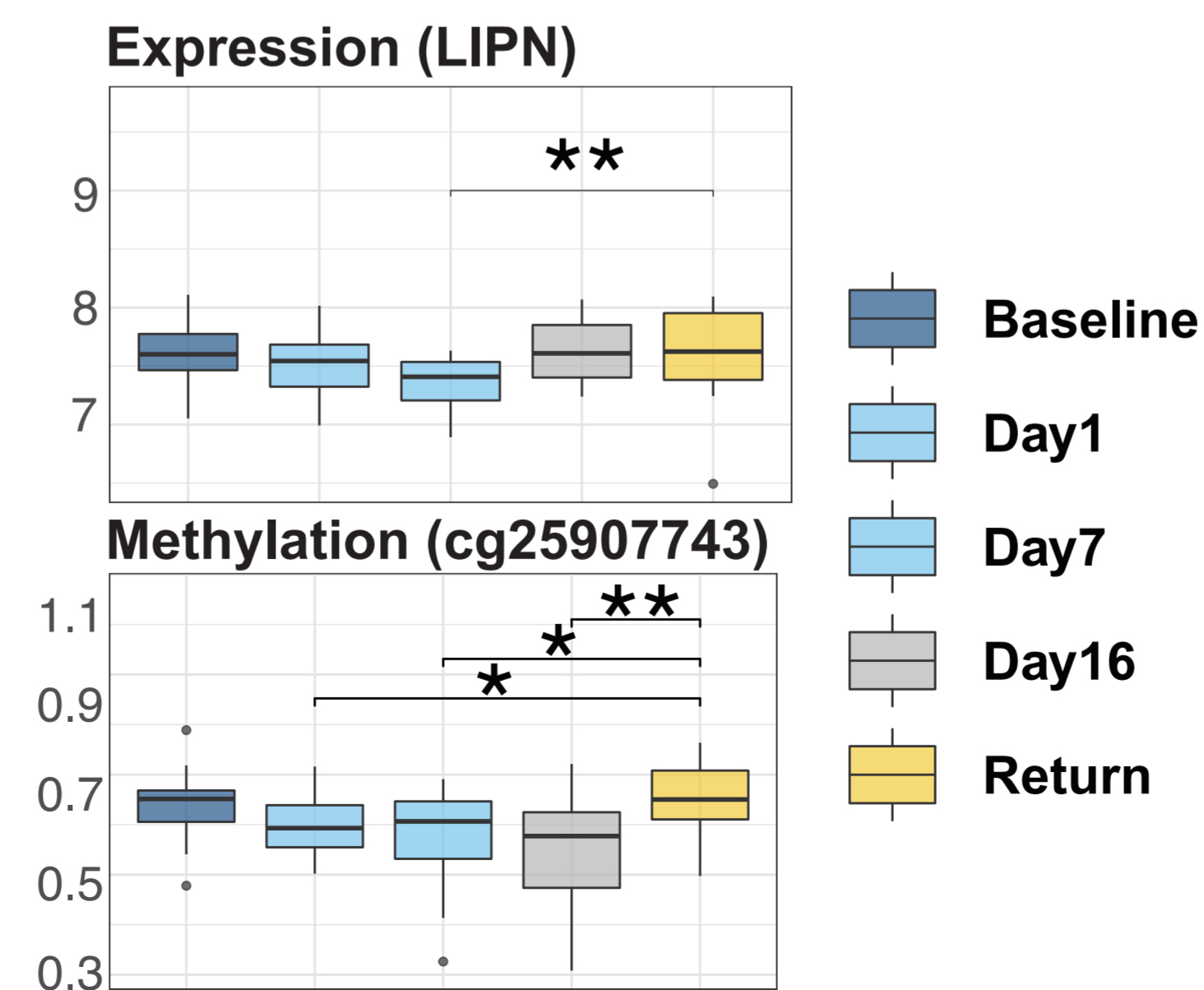

## F STX5 eQTM

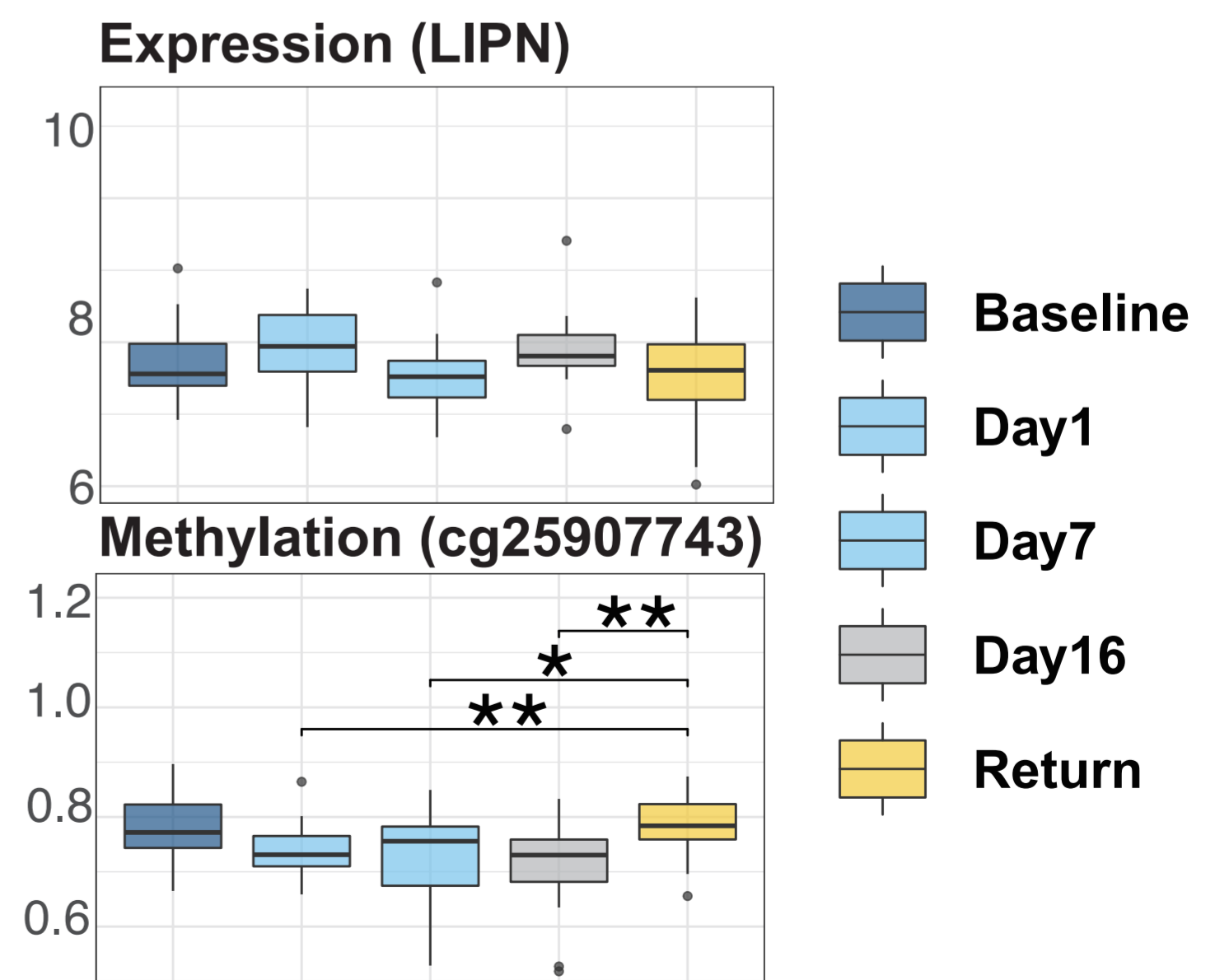

Supplement: Supplementary file 1 [file ijms-25-12652-s001.zip › Fig S2.pdf]

A

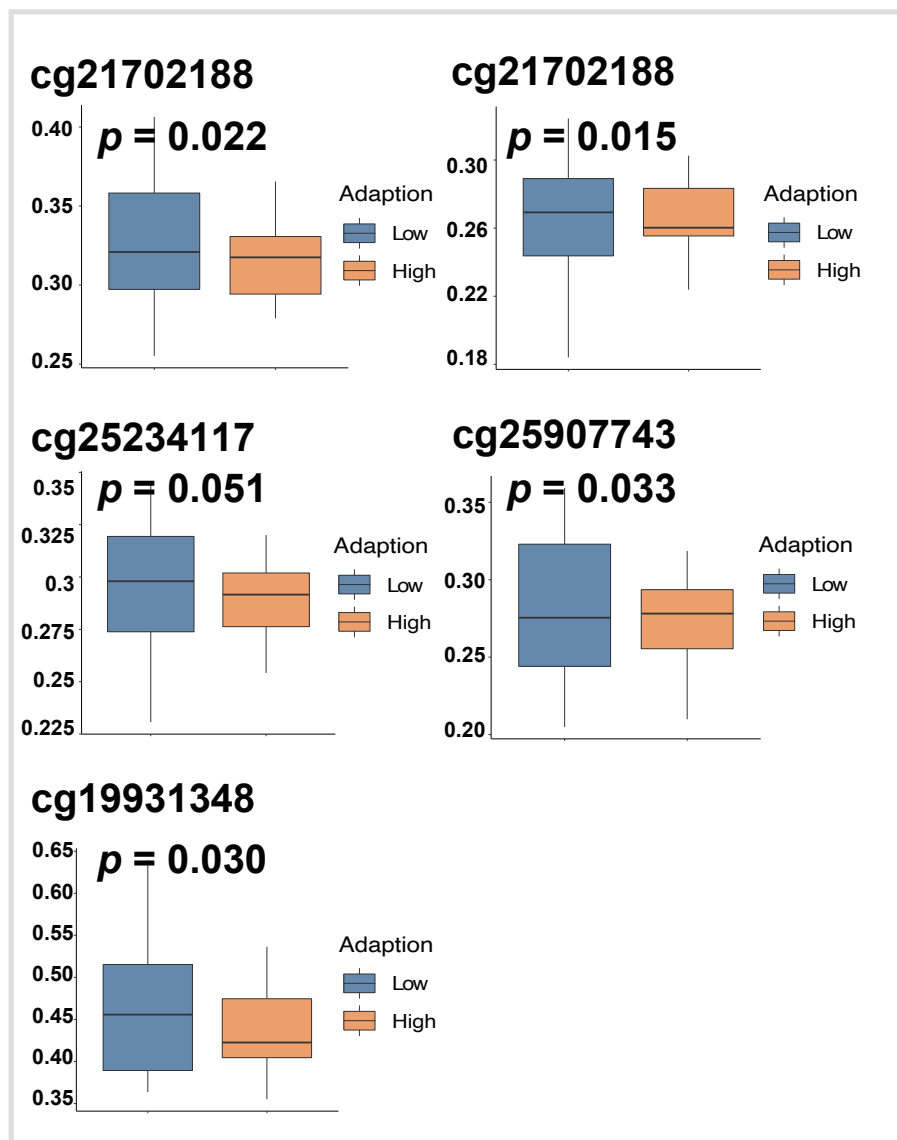

B

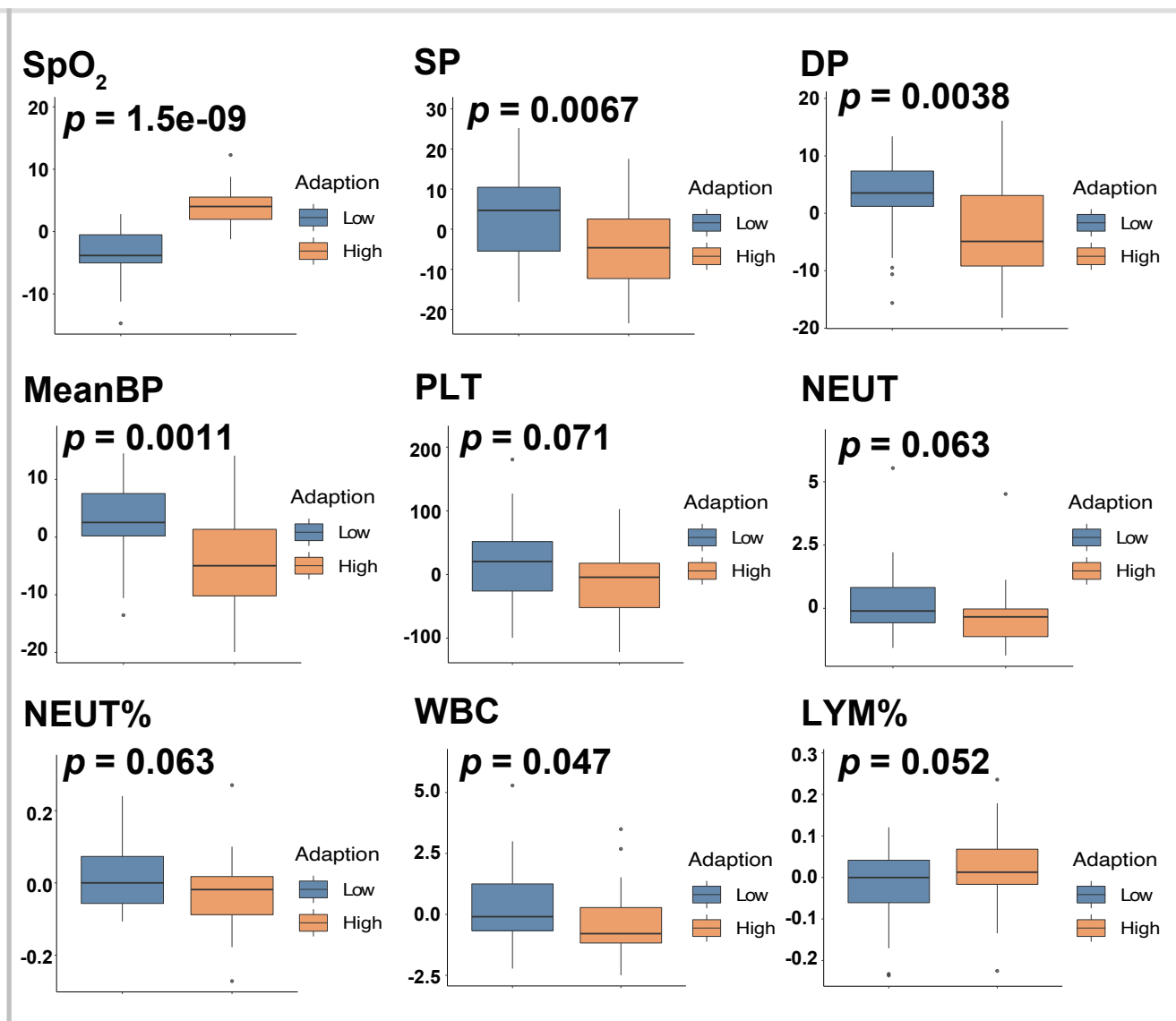

C

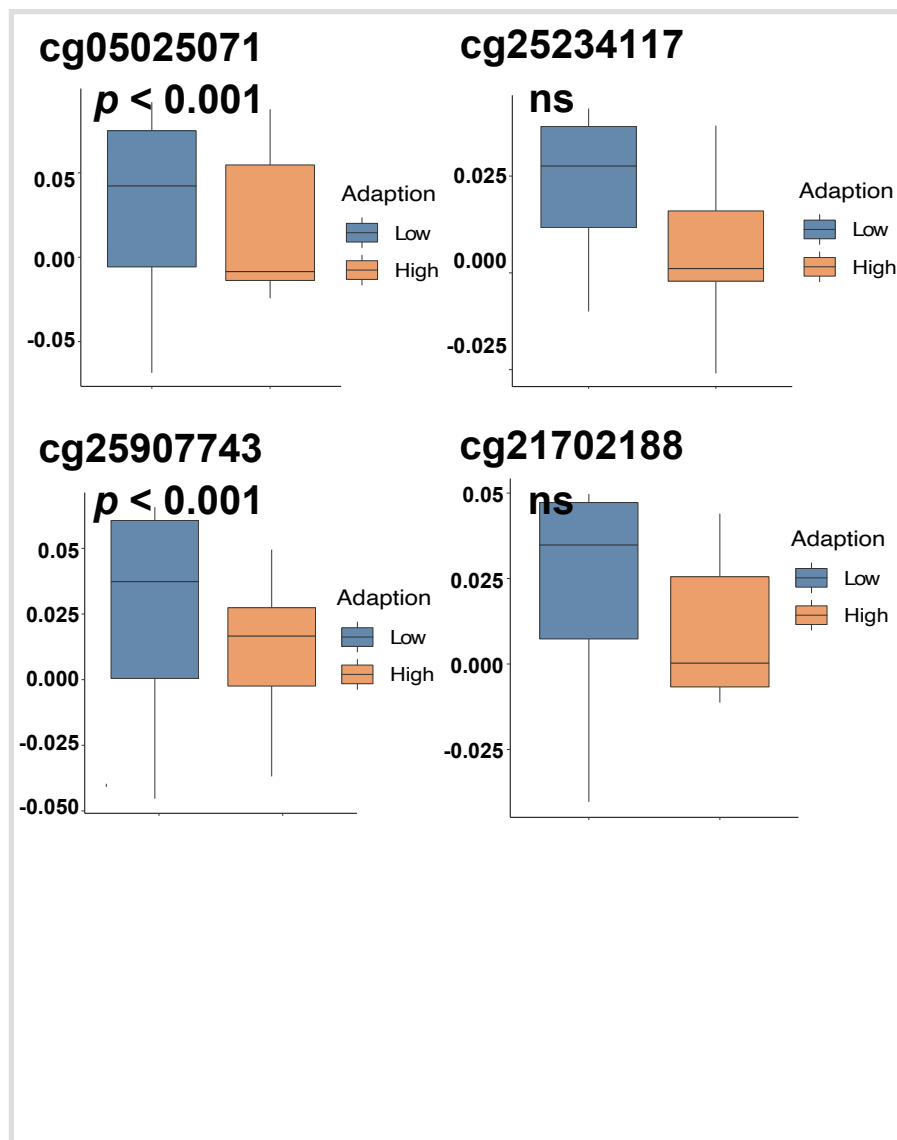

D

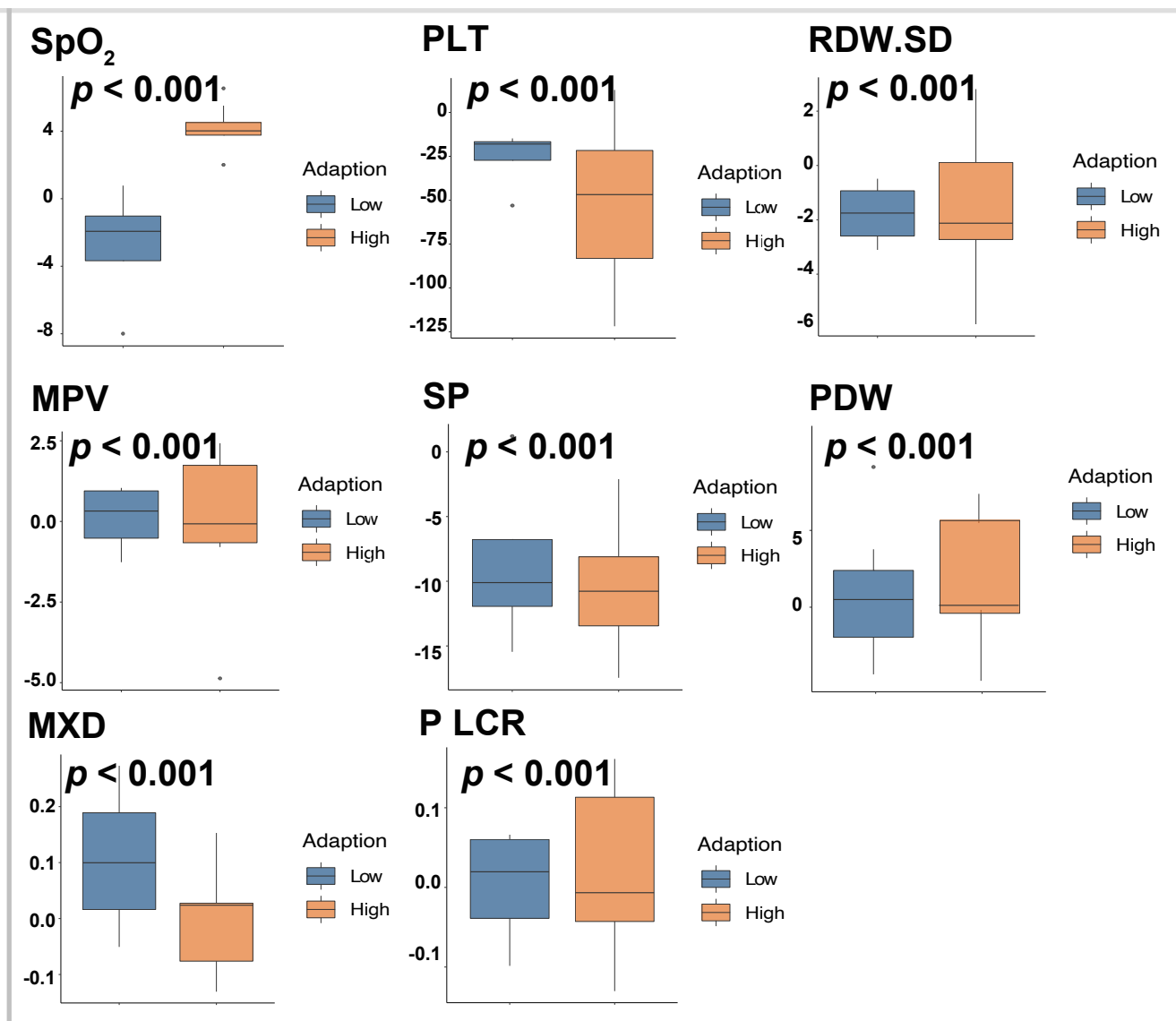

Supplement: Supplementary file 1 [file ijms-25-12652-s001.zip › Fig S3.pdf]

**A****High adaptability**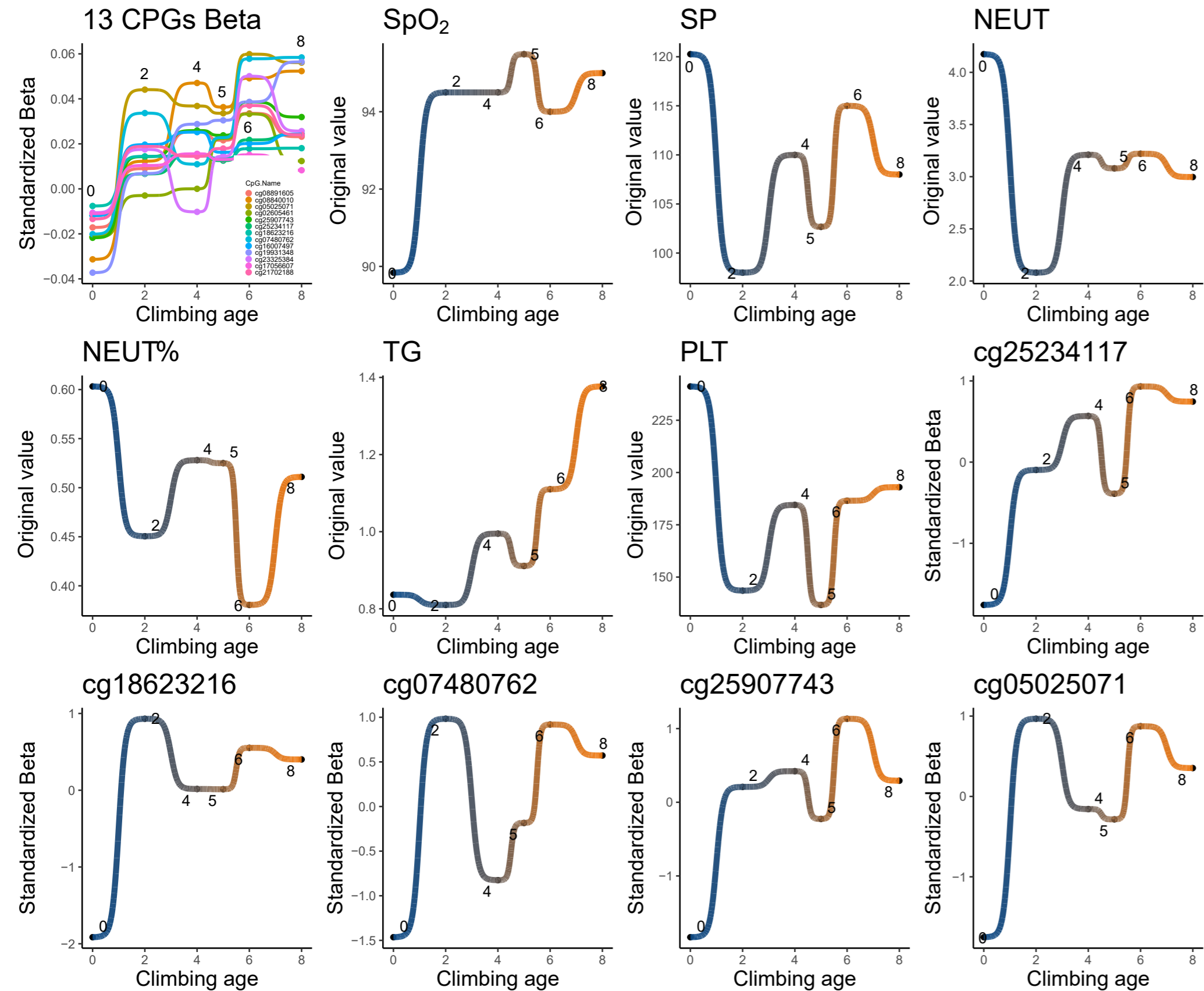**B****Low adaptability**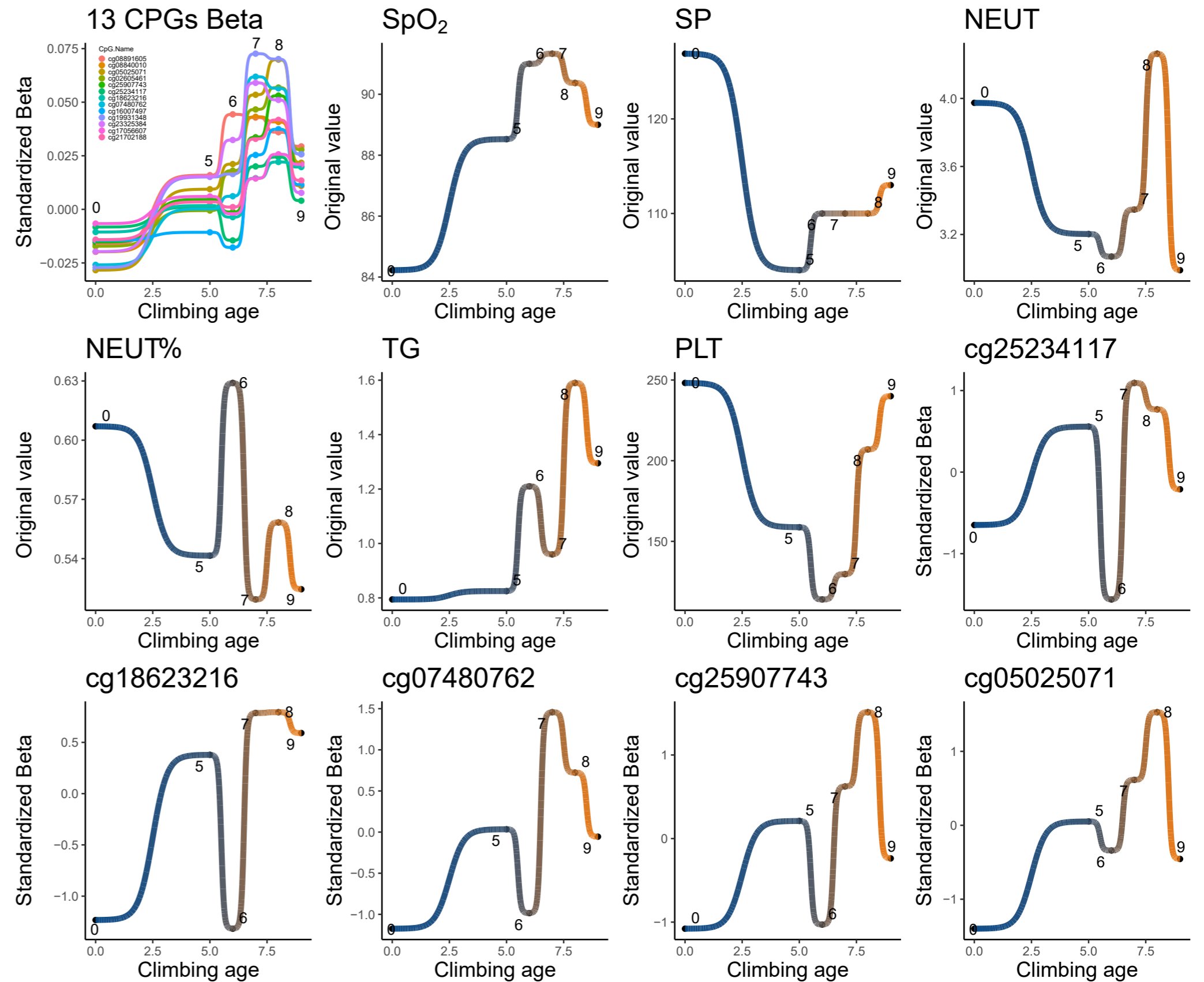

Supplement: Supplementary file 1 [file ijms-25-12652-s001.zip › Fig S4.pdf]

**A**

## Distribution of Non-RETs Cell Infiltration

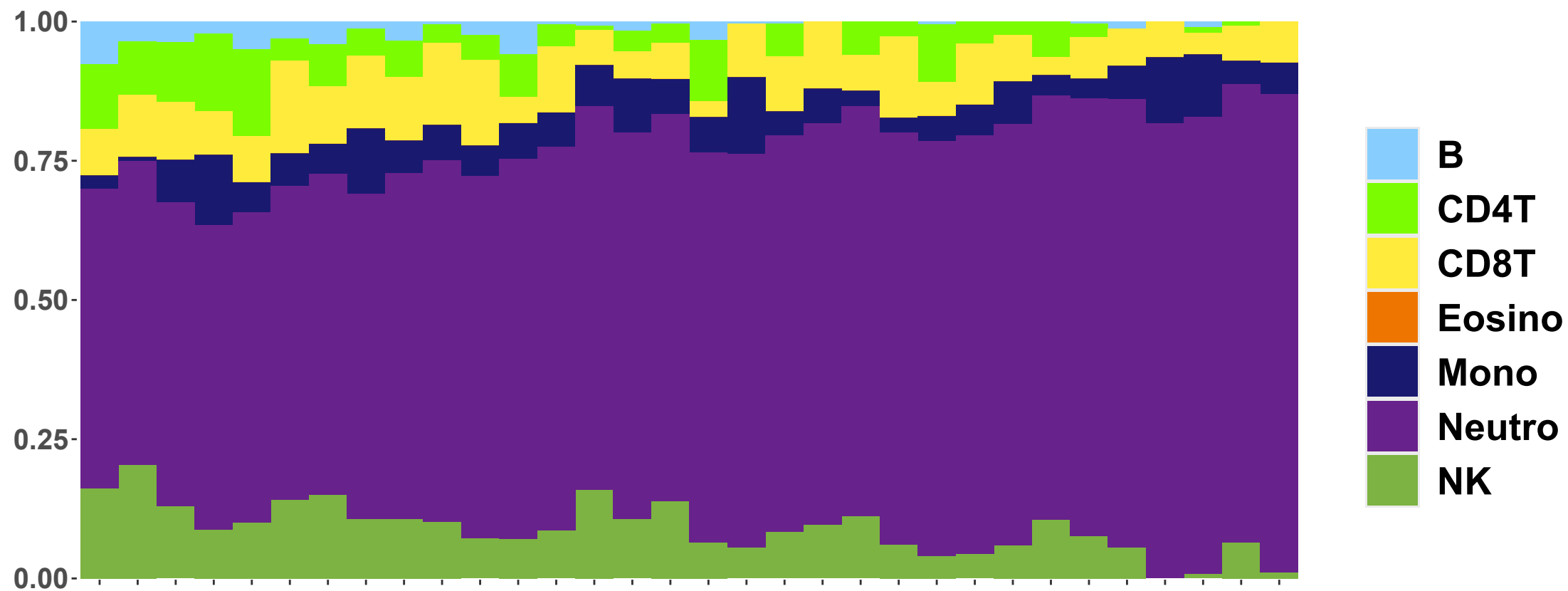

## Distribution of RETs Cell Infiltration

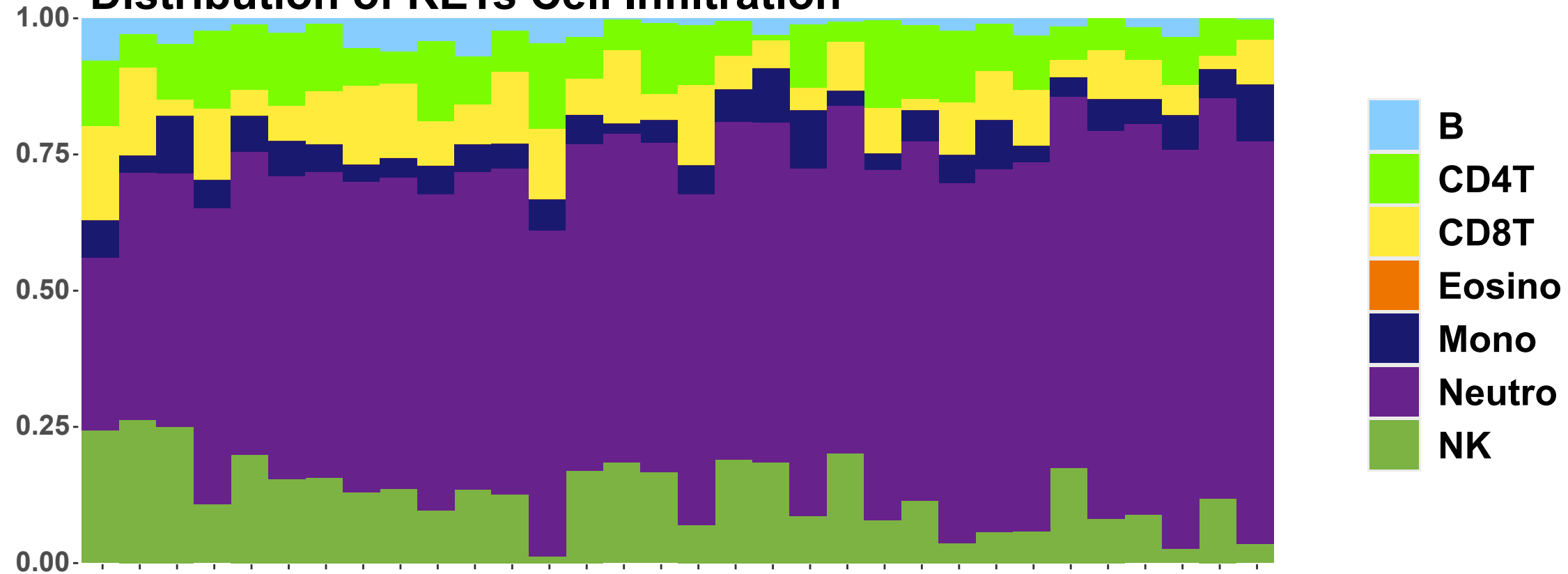**B**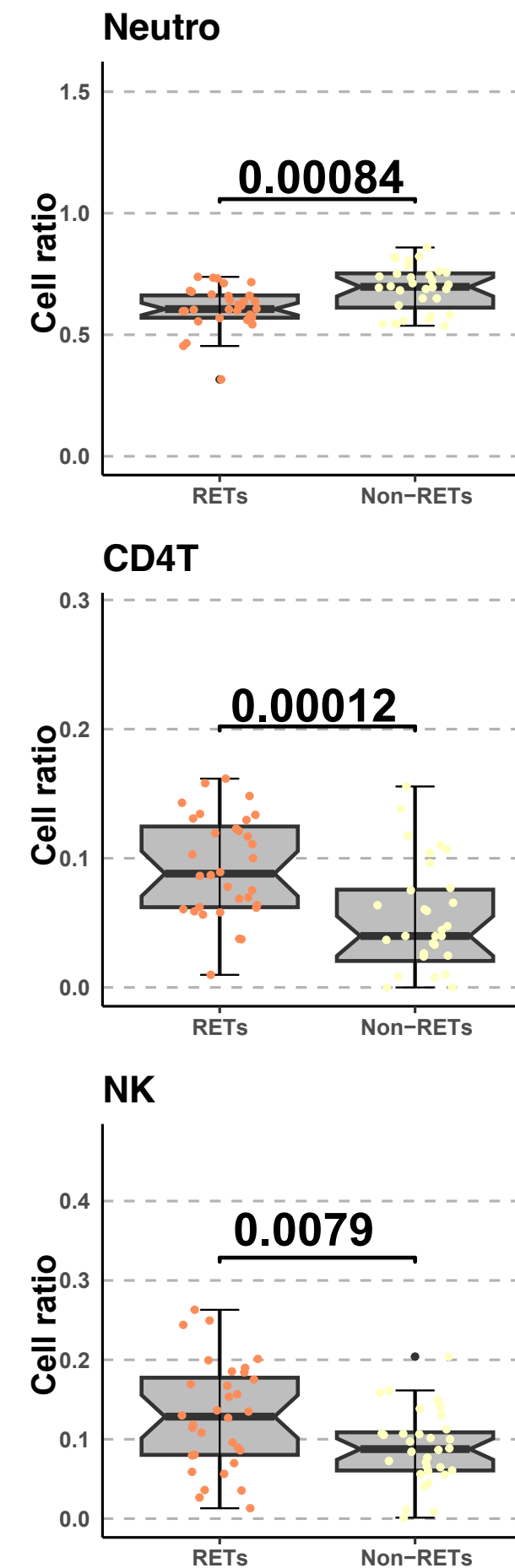

Supplement: Supplementary file 1 [file ijms-25-12652-s001.zip › Fig S5.pdf]
